# Middle East respiratory syndrome coronavirus: transmission, virology and therapeutic targeting to aid in outbreak control

**DOI:** 10.1038/emm.2015.76

**Published:** 2015-08-28

**Authors:** Prasannavenkatesh Durai, Maria Batool, Masaud Shah, Sangdun Choi

**Affiliations:** 1 Department of Molecular Science and Technology, Ajou University, Suwon, Korea

## Abstract

Middle East respiratory syndrome coronavirus (MERS-CoV) causes high fever, cough, acute respiratory tract infection and multiorgan dysfunction that may eventually lead to the death of the infected individuals. MERS-CoV is thought to be transmitted to humans through dromedary camels. The occurrence of the virus was first reported in the Middle East and it subsequently spread to several parts of the world. Since 2012, about 1368 infections, including ~487 deaths, have been reported worldwide. Notably, the recent human-to-human ‘superspreading' of MERS-CoV in hospitals in South Korea has raised a major global health concern. The fatality rate in MERS-CoV infection is four times higher compared with that of the closely related severe acute respiratory syndrome coronavirus infection. Currently, no drug has been clinically approved to control MERS-CoV infection. In this study, we highlight the potential drug targets that can be used to develop anti-MERS-CoV therapeutics.

## Introduction

**Figure 1:**
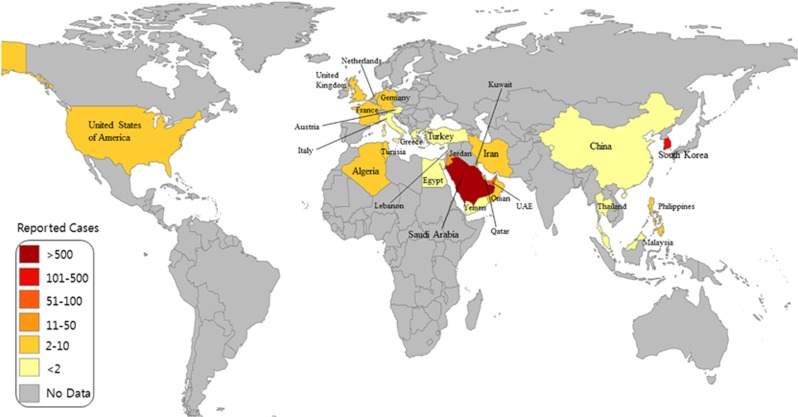
Global distribution map of Middle East respiratory syndrome coronavirus (MERS-CoV). Individuals in 26 countries have been infected by MERS-CoV. The infographic was generated based on MERS-CoV updates released on 7 July 2015 by World Health Organization. (WHO; http://www.who.int/csr/disease/coronavirus_infections/risk-assessment-7july2015/en/).

**Figure 2:**
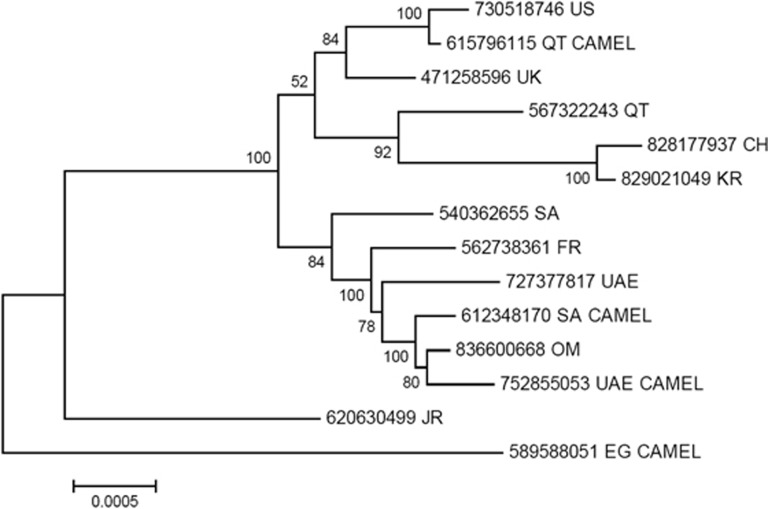
Phylogenetic analysis of the complete Middle East respiratory syndrome coronavirus (MERS-CoV) genomes using the maximum-likelihood method based on Tamura-Nei model implemented in MEGA5. The analysis involved 14 (human and camel) complete MERS-CoV genomes selected from different countries and their accession numbers are given at the end of each branch. The tree was rooted using the Egyptian camel sequence as the most divergent. CH, China, EG, Egypt; FR, France; KR, Republic of Korea; OM, Oman; QT, Qatar; SA, Saudi Arabia, UAE, United Arab Emirates; UK, United Kingdom; US: United States.

In 2012, a new human disease called Middle East respiratory syndrome (MERS), having a high mortality rate, emerged in the Middle East. It was caused by a virus that was originally called human coronavirus-Erasmus Medical Center/2012 (HCoV-EMC/2012), but was later renamed as Middle East respiratory syndrome coronavirus (MERS-CoV).^1^ MERS-CoV is comparable to severe acute respiratory syndrome coronavirus (SARS-CoV), which killed almost 10% of the affected individuals in China between 2002 and 2003.^2^ The first MERS patient reported in Saudi Arabia in June 2012 was possibly infected by direct or indirect transmission of the virus from dromedary camels.^3, 4^ Moreover, MERS-CoV similar to the isolates from dromedary camels and humans was found in bats.^3^ Evidence suggests that MERS-CoV can be transmitted to humans via both animals and humans.^5^ However, the successive epidemics of MERS indicate that the pathogen has spread to various parts of the world predominantly via interhuman transmission (Figure 1). Human-to-human transmission is confirmed by the fact that secondarily infected individuals had come in close contact with a primarily infected individual; these secondarily infected individuals included family members, health-care workers and people who shared the hospital room or visited the patients.^6^ For instance, the first Korean patient affected by MERS-CoV was diagnosed on 20 May 2015 after he returned from Qatar. Owing to the secondary mode of transmission, 186 of Korean citizens have been infected with MERS-CoV in a short span of time. Phylogenetic analysis also suggests that the MERS-CoV isolate found in the Korean patient is closely related to the Qatar strain (Figure 2).

Coronaviruses, members of the *Coronaviridae* family and the *Coronavirinae* subfamily, are found in mammals and birds.^5^ Coronaviruses are divided into four genera: α, β, γ and δ. The human coronaviruses HKU1 (strain named after discovery in the Hong Kong University),^7^ OC43 (labeled with OC because these viruses are grown in ‘Organ Culture'),^8^ SARS-CoV and MERS-CoV belong to the genus β.^9^ SARS-CoV and MERS-CoV are genetically subgrouped into lineages B and C, respectively.^9^ MERS-CoV mainly causes respiratory diseases and systemic disorders.^10^ Gastrointestinal symptoms, including diarrhea and queasiness, are also occasionally observed.^11, 12^ Most MERS-CoV-infected individuals develop chronic comorbidities such as renal failure, diabetes and cardiac disease, resulting in high fatality rates in patients with a history of diabetes and renal failure.^13, 14^ The median age of patients in reported cases is 49 years, and the incubation period ranges between 2 and 13 days, with a median of 5 days.^15^

The physicochemical features of MERS-CoV are listed in Table 1. The MERS-CoV genome is 30 119 nucleotides long and contains 11 open reading frames (ORFs).^16^ The single positive-stranded RNA genome has 5'- and 3'-untranslated regions that are 278 and 300 nucleotides in length, respectively. The 5' end comprises two overlapping ORFs, ORF1a and ORF1b, which are translated to yield two large polyproteins, polyprotein 1a (pp1a) and polyprotein 1ab (pp1ab). These polyproteins are cleaved into 16 functional nonstructural proteins (nsps) by the proteolytic activity of two viral proteases called papain-like protease (PLpro) and 3C-like protease (3CLpro) after their self-cleavage from pp1ab.^11, 17, 18^ Proteolytic processing of MERS-CoV polyproteins is required for the activation of viral replication.^19^ In addition to these two proteases, the two ORFs encode other nsps that are responsible for viral RNA-dependent RNA polymerase activity (nsp12), RNA helicase activity (nsp13), exoribonuclease activity (nsp14), endoribonuclease activity (nsp15) and methyltransferase activity (nsp16).^13^ The role of nsp14 is essential, as it is involved in proofreading by monitoring the mutation rate, a unique feature for an RNA virus.^20^ More genes downstream of ORF1ab encode structural and accessory proteins. Spike (S), envelope (E), membrane (M) and nucleocapsid (N) proteins are all structural proteins, whereas the accessory proteins, unique to this lineage of viruses, are encoded by ORF3, ORF4a, ORF4b, ORF5 and ORF8b.^11^ Although the exact function of these accessory proteins is still unknown, some recent studies have shown that they may have an important role in evading the host immune response.

MERS-CoV enters the host through its S protein, a type I transmembrane glycoprotein with 1353 amino acids (aa) that exists on the virion surface as a trimer.^21^ Subsequently, it is recognized by cluster of differentiation 26 (CD26) (also known as dipeptidyl peptidase 4 (DPP4)), which facilitates the infection of the host cells.^22^ SARS-CoV uses angiotensin-converting enzyme 2 as a functional receptor.^23^ MERS-CoV and SARS-CoV differ in their cellular selection for infection, possibly owing to their selective binding with different receptors.^24^

## Transmission and pathogenesis of MERS-CoV

MERS-CoV infection was initially thought to spread by zoonotic events via bats as phylogenetic studies revealed that it is genetically connected to *Tylonycteris* bat coronavirus HKU4 (BatCoV-HKU4) and *Pipistrellus* bat coronavirus HKU5 (BatCoV-HKU5).^25^ However, evidence indicates that MERS-CoV originated from dromedary camels. A serological study suggests that almost 90% of all camels in Africa and the Middle East were seropositive for MERS-CoV, whereas other animals such as sheep, goats and cows were found to be negative.^3, 5^ A population-based seroepidemiologic study suggests that the seroprevalence of the virus was several folds higher in people who were exposed to camels compared with that in the general population.^3^ Moreover, antibodies against MERS-CoV were found in samples obtained from camels in Saudi Arabia in 1993, which reinforces the hypothesis that dromedary camels are most likely the main reservoirs of MERS-CoV.^14^ In contrast, no seroreactivities were reported in the blood samples obtained from blood donors and abattoir workers in Saudi Arabia during 2012.^14^ MERS-CoV was detected in camels in Egypt that were locally raised or imported from countries where no MERS cases were reported.^14^ The mode of transmission is still unknown but is suspected to be through saliva during direct contact with infected camels or through consumption of milk or uncooked meat. However, we cannot rule out the existence of another intermediate host for MERS-CoV transmission to humans.^3^ Secondary infection may occur through droplets or contact, and the virus could spread either via air or fomites.^13^ A few recent studies on infected patients showed that the most common MERS-CoV infection causes acute pneumonia and renal failure and that almost every patient developed respiratory problems.^26, 27^ In addition, at least one-third of the studied patients were also reported to have abdominal disorders.^27^ Other effects include inflammation of the pericardium, consumptive coagulopathy, increase in leukocytes and neutrophils, and low levels of lymphocytes, platelets and red blood cells.^27^ Moreover, hyponatremia and low blood levels of albumin were detected during the case study.^27^

## Entry and replication of MERS-CoV in the host cell

**Figure 3:**
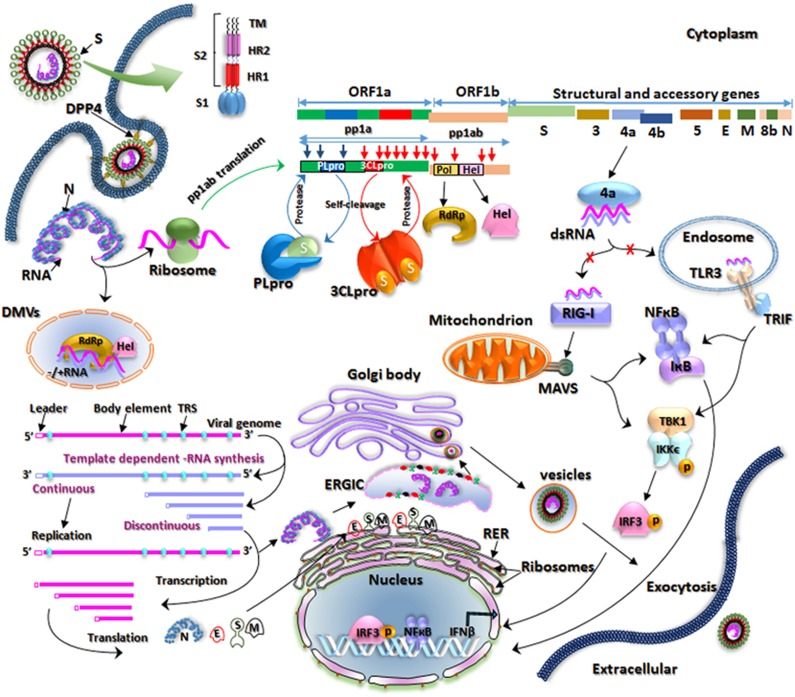
Schematic of the replication cycle of Middle East respiratory syndrome coronavirus (MERS-CoV). MERS-CoV binds to dipeptidyl peptidase 4 (DPP4) on the host cell through its receptor-binding domain (RBD) in the S1 subunit of the spike (S) glycoprotein, which leads to virus–cell fusion and the release of genomic RNA into the cytoplasm. Initially open reading frame 1a (ORF1a) and ORF1b are translated into polyproteins, polyprotein 1a (pp1a) and pp1ab, respectively, which are cleaved by the virus-encoded proteases papain-like protease (PLpro) and 3C-like protease (3CLpro) into 16 mature nonstructural proteins (nsps). The proteins involved in replication and transcription are gathered into replication-transcription complexes (RTCs) that associate with double-membrane vesicles (DMVs) derived from the endoplasmic reticulum (ER). The genomic RNA contains adenylate uridylate (AU)-rich sequences called transcription regulation sequences (TRSs). If the TRSs are recognized by RTCs, then RNA of subgenomic length for transcription will be generated, otherwise a full-length template RNA of genomic length for replication will be synthesized. The newly produced genomic RNAs are encapsidated in the nucleocapsid (N) proteins in the cytoplasm and then transported to the ER–Golgi intermediate compartment (ERGIC) for further assembly. The S, membrane (M) and envelope (E) proteins are inserted into the membrane of the rough ER (RER), from where they are transported to the ERGIC to interact with the RNA-encapsidated N proteins and assemble into viral particles. The budded vesicles containing mature viral particles are then transported to the cell surface for release after maturation in the Golgi bodies. Double-stranded RNAs (dsRNAs) are partially generated during viral replication. The 4a competes with Toll-like receptor 3 (TLR3) and retinoic acid-inducible gene I product (RIG-I)-like helicases (RIG-I and melanoma differentiation-associated protein 5 (MDA5)) to bind to dsRNAs and evades the host immune response.

An overview of the entry and replication process of MERS-CoV in the infected host cell is shown in Figure 3 and is discussed below. The S glycoprotein located on the surface of the MERS-CoV virion interacts with functional receptor DPP4 to facilitate viral entry into the host.^28, 29^ The S protein consists of a globular S1 domain at the N-terminal region; an S2 domain with two heptad repeats (HR), HR1 and HR2; and a transmembrane domain.^30^ The S1 domain determines cell tropism and receptor interaction, whereas membrane-fusing mediators have been identified within the S2 domain.^30, 31^ MERS-CoV binds to DPP4 through a receptor-binding domain (RBD) located in the S1 subunit. Subsequently, protease cleavage of the S protein leads to virus–cell fusion and the release of viral genomic RNA into the host cytoplasm.^32, 33^

The initial translation begins in ORF1a and continues in ORF1b after a frameshift, thereby producing the polyproteins pp1a and pp1ab, respectively.^10, 34^ ORF1ab, which comprises two-thirds of the genome, is responsible for encoding nsps, whereas the remaining one-third of the genome encodes structural proteins (E, N, S and M) and five accessory proteins.^10^ The virus-encoded proteases PLpro and 3CLpro cleave the pp1a and pp1ab proteins at 3 and 11 different sites, respectively, resulting in 16 mature nsps.^9^ The proteins involved in replication and transcription (RNA-dependent RNA polymerase and helicase encoded by ORF1ab) form replication-transcription complexes.^34^ These complexes assemble at the perinuclear regions and associate with double-membrane vesicles derived from the endoplasmic reticulum (ER).^13^ It has been confirmed through electron tomography and three-dimensional reconstruction imaging of the SARS-CoV-infected Vero E6 cells that double-membrane vesicles are not separate vesicles but are instead part of a reticulovesicular system of altered ER membranes.^35^ The genomic RNA contains adenylate uridylate-rich sequences called transcription regulation sequences that are about 10 nucleotides long; these sequences divide the genomic RNA into different body elements of various lengths.^34^ These transcription regulation sequences are either recognized by the replication-transcription complexes to generate discontinued short negative-strand RNA of subgenomic length for the transcription of accessory and structural proteins or continuous full-length minus-strand template RNA of genomic length for replication.^34^

The newly synthesized genomic RNAs are encapsidated in the N proteins in the cytoplasm and then transported to the ER–Golgi intermediate compartment for further assembly.^13, 35^ The proteins S, M and E are inserted into the membrane of the rough ER and are subsequently transported to the ER–Golgi intermediate compartment where they interact with the N proteins and assemble into particles.^35^ The budded vesicles are then transported to the cell surface for release after maturation in Golgi bodies.^13, 35^

In the life cycle of RNA viruses, interferons (IFNs) have a crucial role in the anti-viral defense and are activated by the double-stranded RNAs (dsRNAs) generated during viral replication.^36^ Pattern recognition receptors including Toll-like receptors (TLRs) and retinoic acid inducible gene-I (RIG-I)-like receptors (RIG-I and melanoma differentiation-associated protein 5 (MDA5)) have an essential role in innate immunity.^37, 38, 39^ Among TLRs, TLR3 specifically recognizes dsRNAs through its ecto domain,^40^ whereas RIG-I and MDA5 also recognize dsRNAs through their helicase domain.^37^ After sensing dsRNAs, the activated immune response induces IFNs and cytokines to block viral replication. To evade this cellular immune response, MERS-CoV 4a protein binds to dsRNAs and blocks the induction of type 1 IFN.^41, 42^

## Therapeutic targets

### Spike protein

As we mentioned earlier, the RBD located in S1 subunit binds to DPP4 to initiate infection, and the HR1 and HR2 motifs in S2 subunit facilitate membrane fusion, resulting in the release of the viral genetic material into the host cell cytoplasm.^43^ Based on the crystallographic study, the RBD of the MERS-CoV S1 subunit ranges from residues 367 to 606 and can be divided into a core and an external subdomain.^22^ The receptor-binding motif (V484 to L567) of RBD is located in the external subdomain.^32^ The core subdomain contains a five-stranded antiparallel β-sheet in the center. The six connecting helices and two small β-strands collectively make a globular fold. Three disulfide bonds balance the core domain structure from the internal region. The RBD ends are located close to one another. The external subdomain of MERS-CoV RBD comprises a β-sheet with one small and three large strands organized in an antiparallel manner. It is attached to the RBD core through intervening loops and it attaches to the core subdomain like a clamp at the upper and lower positions. Two small 3_10_ helices and most of the joining loops are present on the inner side of the sheet. The fourth disulfide bond is formed between the C503 and C526 residues, connecting the η3-helix with the β6-strand. Mutational studies have confirmed that residues Y499, L506, W513 and E553 in RBD are required for receptor binding and thus for viral entry.^22, 32^ Mutation of these residues significantly inhibits the interaction of RBD with DPP4. Three HR1 helices at the center and three HR2 chains adjacent to the core in the HR1 side grooves facilitate the release of the viral particles into the cytoplasm.^16, 43^ HR2P (HR2 peptide) that binds to the HR1 domain to block MERS-CoV S protein-induced membrane fusion has been reported.^43^ Moreover, other effective inhibitors that target RBD and could be used to control MERS-CoV infection have recently been reviewed by Xia *et al.*^21^

Two antibodies (REGN3051 and REGN3048) targeting RBD of S protein to prevent its binding to DPP4 were developed and found to be the potential inhibitors of MERS-CoV.^44^ These two antibodies were tested on a mouse model that was developed by substituting mouse DPP4 ORF with human DPP4 (hDPP4) ORF, assuring normal physiological expression of hDPP4. A previously developed animal model was effective but expressed hDPP4 in all types of cells, resulting in non-physiological expression.^45^ In a recent *in vivo* study, modified vaccinia virus Ankara, which stably expresses the MERS-CoV S protein, exhibited less or no MERS-CoV replication.^46^ Moreover, the vaccinated mouse was further infected with MERS-CoV and transduced with hDPP4 to prove its efficacy.^46^

### DPP4/CD26 host receptor

DPP4 is mainly expressed on epithelial cells and controls the activity of hormones and chemokines.^16, 47^ DPP4, a 766-aa-long type-II transmembrane glycoprotein, acts as a unique receptor for MERS-CoV.^26^ Crystallographic study reveals that the DPP4 receptor has an α/β-hydrolase domain and a β-propeller domain with eight blades, where MERS-CoV RBD binds. Small molecules or peptides that prevent the binding of DPP4 and RBD are potential MERS-CoV entry inhibitors and a few have been identified. Adenosine deaminase, a DPP4 binding protein, acts as a competitive inhibitor for MERS-CoV S protein.^48^ An anti-CD26 polyclonal antibody has also shown inhibitory effects on MERS-CoV infection *in vitro*.^49^ A humanized monoclonal antibody against DPP4, mAb YS110, has also been reported to inhibit MERS-CoV infection.^50^ In a recent study, a murine model was developed by transducing a mouse with non-replicating adenovirus that expresses hDPP4.^51^ The transduced mouse developed pneumonia when infected with MERS-CoV. A subunit vaccine (Venezuelan equine encephalitis replicon particles that express MERS-CoV S protein) and an anti-viral drug (poly-I:C) were evaluated by infecting the mouse with MERS-CoV, and the subunit vaccine was found to be effective against the virus. This mouse model can be developed in a period of 2–3 weeks. However, this transduction system regulates hDPP4 expression to a low level and limits the expression to the lungs.

### PLpro and 3CLpro

Processing of the viral polyproteins is necessary for the discharge of mature proteins as they guide the replication and transcription of the MERS-CoV genome.^10^ This is achieved by the two viral proteases PLpro and 3CLpro, located in nsp3 and nsp5, respectively.^10^ These proteases cleave pp1a and pp1ab at several locations.^10^ Initially, both proteases are released in the immature form by the autoproteolytic process. In addition to the role mentioned above, MERS-CoV PLpro also affects ubiquitination and IFN-stimulated gene 15-linked ISGylation, probably to block host anti-viral responses.^52^ MERS-CoV PLpro is able to deubiquitinate IRF3, thereby inhibiting the synthesis of IFNβ.^19^ The MERS-CoV PLpro domain spans residues 1484–1800 in the pp1a protein.^53^ Similar to the PLpro of other coronaviruses, the Cys1592, His1759 and Asp1774 residues of MERS-CoV PLpro coordinate catalysis.^18^ The crystal structure of the MERS-CoV PLpro bound to ubiquitin revealed the interacting amino acids in the active site of PLpro.^18^ In addition, eight different PLpro residues (Arg1649, Thr1653, Ala1656, Asn1673, Val1674, Val1691, Val1706 and Gln1708) were mutated, either individually or in combination, to verify which of them are required for the binding of ubiquitin to MERS-CoV PLpro.^18^ In particular, mutation of Val1691 with Arg had a major effect on deubiquitination. As processing of the polyprotein is essential for viral maturation, MERS-CoV PLpro is considered a promising anti-viral target. In a recent study, a dual non-covalent inhibitor for MERS-CoV PLpro and SARS-CoV PLpro was identified in a high-throughput screening of 25 000 compounds.^9^ The currently available crystal structures and the results of mutational studies on MERS-CoV PLpro will aid in developing new inhibitors.

3CLpro is an essential part of the polyprotein and is usually present as a monomer.^17^ However, upon substrate binding, dimer formation has been observed.^54^ Each monomer has two domains (I and II) along with a C-terminal domain.^54^ 3CLpro is an important drug target, as its protease activity is crucial for viral survival and replication. In a recent study, 11 inhibitors of 3CLpro were identified, two of which were cocrystallized with 3CLpro enzyme.^17^ One of those two peptidomimetic compounds contains a Michael acceptor group and the other has non-covalent properties. The irreversible compounds with a Michael acceptor group prevent the dimerization of 3CLpro in a time-dependent manner. Similarly, non-covalent peptidomimetic compounds inhibit the activity of 3CLpro, but only at high concentrations. Although a limited number of 3CLpro inhibitors are available, the current knowledge of key conserved and non-conserved residues is valuable. The knowledge of interacting residues from the cocrystallized compounds will enable the development of 3CLpro inhibitors that are relatively more effective. A recent study has found that the chloropyridine ester CE-5 inhibitor of SARS-CoV also inhibited the activity of MERS-CoV 3CLpro, reducing it to 30%. This was achieved by transfection of HEK293T cells with 3CLpro-expressing plasmid and evaluation of the protease activity by a luciferase-based biosensor assay.^55^

### Accessory proteins

Each coronavirus has a specific group of genes, which is responsible for encoding accessory proteins.^56^ These accessory proteins do not participate in the structure of MERS-CoV particles but have an essential role in viral replication and evasion of the host immune response.^57, 58, 59, 60, 61^ They are difficult to study because of their low expression level as well as their low molecular weight. In addition, they are not conserved in the coronavirus subfamilies. Although the accessory proteins can be targeted by anti-viral therapeutics, the biological function of these proteins is still not well understood. MERS-CoV has five accessory proteins: 3, 4a, 4b, 5 and 8b, encoded by various ORFs.^56^ The first four accessory proteins are located between the structural proteins S and E, whereas 8b resides downstream of the N protein.^59^ Proteins 3, 4a, 4b, 5 and 8b contain 103, 109, 246, 224 and 112 aa, respectively.

IFNs are secreted by the virus-infected host cells and provide a protective shield to the other exposed cells.^62^ Type 1 IFNs and inflammatory cytokines are produced as a result of the recognition of pathogen-associated molecular patterns by TLRs or RIG-I-like helicases.^63^ The proteins M, 4a, 4b and 5 have been demonstrated to be involved in the inhibition of IFN production and 4a, in particular, strongly affects viral pathogenesis.^56^

Protein 4a, one of the accessory proteins, blocks IFN induction and works as a strong inhibitor of type 1 IFN by inhibiting dsRNA recognition by cellular RIG-I and MDA5.^41, 42^ RIG-I-like helicases (RIG-I and MDA5) recognize dsRNA in the cytoplasm at the time of viral replication and initiate IFN induction through IRF3. RIG-I-like helicases comprise two domains: an RNA-binding domain and a caspase activation and recruitment domain. The dsRNA binds to the RNA-binding domain and induces a conformational change in RIG-I, thereby exposing the caspase activation and recruitment domain. The caspase activation and recruitment domain initiates the downstream signaling, which is detected by the mitochondrial anti-viral signaling adaptor protein, present on the mitochondrial surface. Downstream signaling involves the activation of IRF3, which is phosphorylated and forms a homodimer. The dimer enters the nucleus and initiates the transcription of IFNα and β.^64, 65^ In case of infection with MERS-CoV, infected cells are not able to produce IFN because of the interference of the 4a protein that hinders the binding of dsRNA to RIG-I-like helicases.

Protein 4a is 109 aa long and contains an RNA-binding domain comprising 72 aa. The RNA-binding domain of 4a binds dsRNA and does not allow it to bind to the RNA-binding domain of RIG-I, thereby inhibiting the anti-viral signaling pathway. Thus, the virus blocks the innate immune response and continues infecting cells. The two key residues involved in the binding of RNA to the RNA-binding domain in 4a are K63 and K67.^42^ Inhibition of the 4a protein can allow the host cell to initiate an immune response against the virus.

## Other therapeutics

With reference to other therapeutic findings for SARS-CoV, the possibilities and findings on MERS-CoV subunit vaccines have been reviewed by Zhang *et al.*^16^ Mycophenolic acid, cyclosporin A, IFNα and IFNβ effectively inhibit MERS-CoV replication.^66, 67, 68^ Ribavirin has previously been used against SARS-CoV but has also been found to control MERS-CoV.^66^ Moreover, the efficiency of ribavirin against MERS-CoV was increased when cotreated with IFNα2b.^66^ A collection of 27 dual inhibitors for MERS-CoV and SARS-CoV were selected from a list of 290 compounds through an *in vitro* study using the MERS-CoV Jordan strain.^69^ Additionally, SSYA10-001 was found to inhibit MERS-CoV replication when tested using the same strain.^70^ In a similar study, four Food and Drug Administration-approved drugs, loperamide, chlorpromazine, lopinavir and chloroquine, were identified to inhibit MERS-CoV replication at micromolar concentrations.^71^ In addition, K22, a small molecule that inhibits membrane-bound MERS-CoV replication, was identified by screening 16 671 compounds.^72^ The MERS-CoV E protein, which is involved in viral assembly, budding and intracellular trafficking, can be targeted for anti-viral activity.^73^ The *in vitro* studies have a major role to confirm the initial anti-viral findings and thus several cell lines and their suitability for MERS-CoV transfection were reported; this information is summarized in Table 2.^74, 75, 76, 77, 78^

## Concluding remarks

MERS-CoV persists as a life-threatening disease. This coronavirus has rapidly evolved and MERS has emerged as a global pandemic. Despite the research efforts undertaken so far, the exact intermediate host for MERS-CoV and spatial distribution are still not well known. In addition, data on the origin and evolution of MERS-CoV are lacking. Global health concerns about this virus are increasing, and effective anti-MERS-CoV drugs and vaccines have yet to be developed and approved. The proteins involved in MERS-CoV entry and replication are attractive targets for the development of anti-viral therapeutics. The available crystal structures of the viral structural, nonstructural and accessory proteins and understanding the binding mechanism of their reported inhibitors may help to develop effective anti-MERS-CoV drugs. Development of suitable murine models and availability of drug-testing techniques have sped up the identification of new drugs and the confirmation of their anti-viral efficacy. Companies are not eager to develop effective vaccines or drugs considering the lack of commercial benefits from their sales. However, to avoid another unexpected global pandemic in the future, it is necessary to develop effective therapies. If this initiative cannot be taken by profit-pursuing companies, it must be carried forward by the governments of developed countries or by philanthropic scientists.

## Figures and Tables

**Table 1:** Physicochemical features of MERS-CoV proteins

*Protein*	*GenBank ID (protein)*	*Start position (nt)*	*End position (nt)*	*Length (aa)*	*MW*^a^	*Stability*^a^	*SP*^b^	*Crystal structure*
PL protease	AGV08556	4728	5678	317	35.9	Stable	No	Yes
3CL protease	YP_009047217	10 020	10 937	306	33.3	Stable	No	Yes
RdRp	YP_009047223	13 410	16 202	933	106.9	Stable	No	No
Helicase	YP_009047224	20 606	21 493	598	66.1	Stable	No	No
Spike	AKL59401	21 456	25 517	1353	149.4	Stable	Yes	Yes
ORF3	AKL59402	25 532	25 843	103	11.2	Stable	Yes	No
ORF4a	AKL59403	25 851	26 180	109	12.2	Unstable	No	No
ORF4b	AKL59404	26 092	26 832	246	28.5	Unstable	No	No
ORF5	AKL59405	26 839	27 513	224	25.2	Unstable	No	No
Envelope	AKL59406	27 590	27 838	82	9.3	Stable	No	No
Membrane	AKL59407	27 853	28 512	219	24.5	Unstable	No	No
Nucleoprotein	AKL59408	28 566	29 807	413	45.0	Unstable	No	No
ORF8b	AJD81448	28 718	29 056	112	12.2	Unstable	No	No

Abbreviations: aa, amino acid; MW, molecular weight; nt, nucleotide; ORF, open reading frame; PL, papain like; RdRp, RNA-dependent RNA polymerase; SP, signal peptides.

a The online tool Protparam is used for the prediction of the MW and stability of proteins.

b The online tool SignalP is used to predict the presence of SPs in the proteins.

**Table 2:** Cell lines and their suitability for MERS-CoV transfection

*No.*	*Cell line*	*MERS-CoV transfection*	*References*
1	Human primary bronchial epithelial cells	Yes	^75^
2	Mouse embryonic fibroblasts (NIH-3T3)	No	^74, 77^
3	Human primary kidney epithelial cells	Yes	^75, 77^
4	Porcine kidney epithelial cells (PK-15)	Yes	^74, 76, 77^
5	Rat kidney mesangium cells (RMC)	No	^74, 77^
6	Chicken fibroblasts (DF-1)	No	^74, 77^
7	Human kidney cancer cells (769-P)	Yes	^76^
8	Human alveolar adenocarcinoma epithelial cells (A549)	Yes	^74^
9	Bat kidney cells (*Rousettus aegyptiacus*, RoNi/7; *Pipistrellus pipistrellus*, PipNi/1 and PipNi/3; *Carollia perspicillata*, CarNi/1)	Yes	^76, 78^
10	Insect *Aedes albopictus* cells (C6-36)	No	^74, 77^
11	Bat lung epithelial cells (*Rhinolophus landeri*, RhiLu; *Myotis daubentonii*, MyDauNi/2)	Yes	^76, 78^
12	Goat lung primary epithelial cells (ZLu-R)	Yes	^74, 78^
13	Alpaca kidney epithelial cells (LGK-1-R)	Yes	^74, 78^
14	Dromedary umbilical cord cells (TT-R.B)	Yes	^74^
15	Baby hamster kidney epithelial cells (BHK)	No	^74, 76^
16	African green monkey kidney epithelial cells (MA104)	No	^74, 76^
17	Madin-Darby canine kidney epithelial cells (MDCK)	No	^74, 77^
18	Feline kidney epithelial cells (CRFK)	No	^74, 77^
19	Rabbit kidney epithelial cells (RK-13)	No	^74, 77^
20	Human colorectal adenocarcinoma cell line (Caco-2)	Yes	^74^
21	Human hepatocellular carcinoma cell line (Huh-7)	Yes	^74, 77^

Abbreviation: MERS-CoV, Middle East respiratory syndrome coronavirus.
